# Supplementary material for: Estimating the effect of tracking tag weight on insect movement using video analysis: A case study with a flightless orthopteran
Source: PLoS One. 2021 Jul 22;16(7):e0255117. doi: 10.1371/journal.pone.0255117 (PMC8297838; doi:10.1371/journal.pone.0255117)
Supplement: S1 Table — P-values of Mann-Whitney tests of movement properties of control crickets and crickets carrying light, medium, or heavy tags with and without regard to temperature. (PDF) [file pone.0255117.s001.pdf]

S1 Table. Tests for the effect of tag weight on the movement properties of crickets. P-values of Mann-Whitney tests of movement properties of control crickets and crickets carrying light, medium, or heavy tags with and without regard to temperature.

A - P-values of Mann-Whitney tests of movement properties of control crickets and crickets carrying light, medium, or heavy tags without regard to temperature. Gray highlighted values indicate significant results ($p < 0.05$)

	control vs. light	control vs. medium	control vs. heavy
Day 1-movementLength	0.357	0.403	0.218
Day 2-movementLength	0.234	0.280	0.218
Day 3-movementLength	0.534	0.327	0.048
Day 1-movementLengthMax	0.364	0.046	0.023
Day 2-movementLengthMax	0.356	0.016	0.012
Day 3-movementLengthMax	0.722	0.068	0.014
Day 1-movementSum	0.234	0.048	0.003
Day 2-movementSum	0.015	0.001	<0.001
Day 3-movementSum	0.620	0.014	<0.001
Day 1-movementSpeed	0.023	0.006	<0.001
Day 2-movementSpeed	0.012	<0.001	<0.001
Day 3-movementSpeed	0.114	0.001	<0.001
Day 1-restingDuration	0.862	0.357	0.107
Day 2-restingDuration	0.195	0.039	0.000
Day 3-restingDuration	0.168	0.356	0.048
Day 1-restingFrequency	0.031	0.395	0.319
Day 2-restingFrequency	0.807	0.862	0.573
Day 3-restingFrequency	0.048	0.339	0.364

B - P-values of Mann-Whitney tests for the effect of temperature (low, intermediate, and high) on movement properties of crickets carrying light, medium, or heavy tags relative to those carrying no tag (control). Gray highlighted values indicate significant results ($p < 0.05$)

	low			intermediate			high		
	light	medium	heavy	light	medium	heavy	light	medium	heavy
Day 1-movementLength	0.010	0.060	<0.001	0.800	0.920	0.780	0.850	0.920	0.940
Day 2-movementLength	0.030	0.030	<0.001	0.920	0.920	0.960	0.920	1.000	0.790
Day 3-movementLength	<0.001	0.030	0.010	0.450	0.700	0.920	0.910	0.930	0.790
Day 1-movementLengthMax	0.160	<0.001	<0.001	0.850	0.450	0.360	0.850	0.930	0.920
Day 2-movementLengthMax	0.140	0.010	<0.001	0.790	0.800	0.780	0.920	0.780	0.930
Day 3-movementLengthMax	0.120	<0.001	<0.001	0.450	1.000	0.850	0.930	0.940	0.640
Day 1-movementSum	0.050	<0.001	<0.001	0.670	0.790	0.090	0.900	0.960	0.850
Day 2-movementSum	<0.001	<0.001	<0.001	0.830	0.580	0.200	0.790	0.850	0.100
Day 3-movementSum	<0.001	<0.001	<0.001	0.270	0.640	0.040	0.580	0.930	0.570
Day 1-movementSpeed	0.090	<0.001	<0.001	0.620	0.630	0.040	0.360	0.620	0.030
Day 2-movementSpeed	<0.001	<0.001	<0.001	0.600	0.110	0.010	0.600	0.410	0.010
Day 3-movementSpeed	0.010	<0.001	<0.001	0.990	0.100	<0.001	0.800	0.570	0.030
Day 1-restingLength	0.900	0.070	0.010	0.930	0.920	0.630	0.410	0.790	0.790
Day 2-restingLength	0.130	<0.001	<0.001	0.760	0.760	0.470	0.940	0.880	0.170
Day 3-restingLength	0.900	0.040	0.010	0.390	1.000	0.760	0.100	0.910	0.790
Day 1-restingFrequency	0.030	0.600	0.930	0.900	0.930	0.210	0.240	0.570	0.630
Day 2-restingFrequency	0.440	0.630	0.580	0.640	0.990	0.920	0.850	0.900	0.930
Day 3-restingFrequency	0.140	0.620	0.850	0.930	0.780	0.800	0.130	0.790	0.290
