# Supplementary material for: Estimating the effect of tracking tag weight on insect movement using video analysis: A case study with a flightless orthopteran
Source: PLoS One. 2021 Jul 22;16(7):e0255117. doi: 10.1371/journal.pone.0255117 (PMC8297838; doi:10.1371/journal.pone.0255117)
Supplement: S2 Table — P-values of Mann-Whitney tests for the effect of temperature on movement properties of crickets separately for each weight category (light, medium, heavy) and for control crickets. (PDF) [file pone.0255117.s002.pdf]

S2 Table. Effect of temperature on movement properties for crickets in separate tag weight categories and for control crickets. P-values of Mann-Whitney tests for the effect of temperature on movement properties of crickets separately for each weight category (light, medium, heavy) and for control crickets.

A - P-values of Mann-Whitney tests for the effect of temperature on movement properties of control crickets. Gray highlighted values indicate significant results ($p < 0.05$)

	low vs. intermediate	low vs. high	intermediate vs. high
Day 1-movementLength	0.010	<0.001	0.059
Day 2-movementLength	0.008	<0.001	0.200
Day 3-movementLength	0.006	<0.001	0.235
Day 1-movementLengthMax	0.034	<0.001	0.040
Day 2-movementLengthMax	0.037	0.001	0.383
Day 3-movementLengthMax	0.003	<0.001	0.314
Day 1-movementSum	0.033	<0.001	0.013
Day 2-movementSum	0.100	0.020	0.871
Day 3-movementSum	0.125	0.007	0.125
Day 1-movementSpeed	0.687	0.383	0.314
Day 2-movementSpeed	0.537	0.450	1.000
Day 3-movementSpeed	0.190	0.287	0.919
Day 1-restingDuration	0.034	<0.001	0.008
Day 2-restingDuration	0.084	0.006	0.495
Day 3-restingDuration	0.049	0.005	0.071
Day 1-restingFrequency	0.885	0.013	0.010
Day 2-restingFrequency	0.647	0.537	0.345
Day 3-restingFrequency	0.885	0.228	0.200

B - P-values of Mann-Whitney tests for the effect of temperature on movement properties of crickets with light tag. Gray highlighted values indicate significant results ($p < 0.05$)

	low vs. intermediate	low vs. high	intermediate vs. high
Day 1-movementLength	0.902	0.018	0.059
Day 2-movementLength	0.714	0.239	0.250
Day 3-movementLength	0.335	0.310	0.102
Day 1-movementLengthMax	0.714	0.335	0.543
Day 2-movementLengthMax	0.802	0.390	0.202
Day 3-movementLengthMax	0.732	0.229	0.098
Day 1-movementSum	0.593	0.038	0.163
Day 2-movementSum	0.543	0.714	0.390
Day 3-movementSum	0.038	0.919	0.065
Day 1-movementSpeed	0.220	0.902	0.065
Day 2-movementSpeed	0.006	0.013	1.000
Day 3-movementSpeed	0.001	0.001	0.406
Day 1-restingDuration	0.310	0.013	0.310
Day 2-restingDuration	0.335	0.323	0.904
Day 3-restingDuration	0.335	0.202	0.335
Day 1-restingFrequency	0.164	0.065	0.500
Day 2-restingFrequency	0.310	0.220	0.902
Day 3-restingFrequency	0.269	0.745	0.335

C - P-values of Mann-Whitney tests for the effect of temperature on movement properties of crickets with medium tag. Gray highlighted values indicate significant results ($p < 0.05$)

	low vs. intermediate	low vs. high	intermediate vs. high
Day 1-movementLength	0.261	0.013	0.241
Day 2-movementLength	0.940	0.241	0.241
Day 3-movementLength	0.638	0.355	0.127
Day 1-movementLengthMax	0.445	0.261	0.440
Day 2-movementLengthMax	0.521	0.890	0.355
Day 3-movementLengthMax	0.355	0.940	0.355
Day 1-movementSum	0.445	0.261	0.200
Day 2-movementSum	0.308	0.355	0.967
Day 3-movementSum	0.159	0.838	0.200
Day 1-movementSpeed	0.005	0.261	0.207
Day 2-movementSpeed	0.005	0.011	0.728
Day 3-movementSpeed	<0.001	<0.001	0.890
Day 1-restingDuration	0.967	0.203	0.203
Day 2-restingDuration	0.838	0.967	0.856
Day 3-restingDuration	0.838	0.272	0.241
Day 1-restingFrequency	0.904	0.203	0.355
Day 2-restingFrequency	0.838	0.200	0.203
Day 3-restingFrequency	0.967	0.316	0.440

D - P-values of Mann-Whitney tests for the effect of temperature on movement properties of crickets with heavy tag. Gray highlighted values indicate significant results ($p < 0.05$)

	low vs. intermediate	low vs. high	intermediate vs. high
Day 1-movementLength	0.503	0.291	0.503
Day 2-movementLength	0.900	0.436	0.338
Day 3-movementLength	0.986	0.401	0.291
Day 1-movementLengthMax	0.728	0.401	0.291
Day 2-movementLengthMax	0.565	0.986	0.728
Day 3-movementLengthMax	0.943	0.565	0.291
Day 1-movementSum	1.000	0.401	0.291
Day 2-movementSum	0.401	0.900	0.550
Day 3-movementSum	0.291	0.963	0.401
Day 1-movementSpeed	0.503	0.986	0.550
Day 2-movementSpeed	0.021	0.021	0.986
Day 3-movementSpeed	<0.001	0.021	0.900
Day 1-restingDuration	0.655	0.565	0.188
Day 2-restingDuration	0.653	0.943	0.495
Day 3-restingDuration	0.653	0.986	0.565
Day 1-restingFrequency	0.401	0.550	0.021
Day 2-restingFrequency	0.565	0.986	0.401
Day 3-restingFrequency	0.653	0.646	0.986
