# Supplementary material for: Estimating the effect of tracking tag weight on insect movement using video analysis: A case study with a flightless orthopteran
Source: PLoS One. 2021 Jul 22;16(7):e0255117. doi: 10.1371/journal.pone.0255117 (PMC8297838; doi:10.1371/journal.pone.0255117)
Supplement: S3 Table — (PDF) [file pone.0255117.s003.pdf]

S3 Table. Effects of the indicated variables on the distance traveled *movementSum* by crickets over 3 days.

	Df	Sum Sq	Mean Sq	F value	Pr(>F)
temp	2	1328.07	664.03	20.02	<0.001
dummy_w	1	3096.34	3096.33	93.34	<0.001
anim_w	1	1626.08	1626.08	49.02	<0.001
temp:dummy_w	2	930.46	465.23	14.03	<0.001
temp:anim_w	2	230.23	115.12	3.47	0.0318
dummy_w:anim_w	1	3.47	3.47	0.10	0.7465
Residuals	524	17381.58	33.17		
