# Supplementary material for: Estimating the effect of tracking tag weight on insect movement using video analysis: A case study with a flightless orthopteran
Source: PLoS One. 2021 Jul 22;16(7):e0255117. doi: 10.1371/journal.pone.0255117 (PMC8297838; doi:10.1371/journal.pone.0255117)
Supplement: S4 Table — Medians and Wilcoxon test of differences in distributions of slopes of linear models that describe change in movement properties as affected by tag weight. (PDF) [file pone.0255117.s004.pdf]

S4 Table. Change in movement properties during 3 consecutive days. Medians and Wilcoxon test of differences in distributions of slopes of linear models that describe change in movement properties as affected by tag weight.

A - Medians of linear models slopes that describe change in monitored properties as affected by tag weight. Gray highlighted values indicate significant Wilcoxon tests ($p < 0.01$) of differences in distributions of slopes relative to control crickets.

	control	light	medium	heavy
movementLength	0.004	0.004	0.002	0.002
movementLengthMax	0.015	0.036	0.026	-0.00002
movementSum	1.695	1.553	1.246	0.356
movementSpeed	0.001	0.003	0.0005	-0.0005
restingDuration	-0.078	-0.251	-0.174	-0.183
restingFrequency	2.000	3.000	1.000	0.500

B - P-values of Wilcoxon test of differences in distributions of slopes of linear models that describe the change in properties as affected by tag weight. Gray highlighted values indicate significant result ($p < 0.01$)

	control vs. light	control vs. medium	control vs. heavy
movementLength	0.676	0.797	0.268
movementLengthMax	0.227	0.531	0.816
movementSum	0.460	0.766	0.231
movementSpeed	0.910	0.120	0.009
restingDuration	0.288	0.624	0.836
restingFrequency	0.802	0.678	1.000
