# Supplementary material for: Estimating the effect of tracking tag weight on insect movement using video analysis: A case study with a flightless orthopteran
Source: PLoS One. 2021 Jul 22;16(7):e0255117. doi: 10.1371/journal.pone.0255117 (PMC8297838; doi:10.1371/journal.pone.0255117)
Supplement: Attachment — Submitted filename: PONE-D-21-05023_reviewercomments.docx [file pone.0255117.s005.docx]

Summary:

In this paper, the authors describe a method to use video analysis to determine if there is an impact of tag weight and environment temperature on insect movement. The authors conclude that effects of tag weight are species-specific and could depend on environmental conditions.

This is a great approach!!! This method will speed up the process of determining if insects are impacted by the attachment of a tracking tag. There are really great aspects of this research! However, it would benefit from a rewrite keeping in mind WHY the study was conducted. It is currently vague and disjointed. I would like more justification for decisions made and a more descriptive description of the results. The part about temperature is not clearly described – I am left wondering why temperature was included as a treatment and not just as “we maintained temperature between x and y degrees Celsius because those are typical conditions”

Introduction

Overall the introduction could benefit from more specific information. Currently, it reads like a brief overview and is not as compelling as it would be with specific examples and information. Why is this important? What makes this study unique? What methodological problem does this solve?

1^st^ paragraph (lines 2-20) – Give an estimate of what you mean by light and heavy radio telemetry tags. How heavy are heavy tags and how long do their batteries last? How light are light tags and how long do their batteries last? What do you mean by smaller animals? This paragraph overall feels vague and would benefit from more specific examples from the literature.

2^nd^ paragraph (lines 21-36) – This paragraph changes topic from how studies account for tag attachment to the impact of temperature to tags outside of radio telemetry and then finished with vertebrates. The ideas are good! But the flow is confusing. Could benefit from splitting into 2 or several paragraphs. Remember the 1^st^ sentence of the paragraph should act as a topic sentence for the entire paragraph. – Maybe one paragraph on the lack of substantial data on tag effects on animals (insects and vertebrates) and how it could be for any type of tag, not just radio telemetry. Then a paragraph on the influence of temperature of insect movement and how it could relate to tag attachment.

3^rd^ paragraph (lines 37-46) – Add a little more justification for why you think different weights and different temperatures will influence movement. Also, what is the justification of the effect changing over time? Justification of affecting cricket body mass?

Methods

1.1 Model species: Be more specific with details. What is their range in size rather than just saying “sufficient size for the experiment”. What was provided as food? How many crickets were in each container? Why did you use 2^nd^ generation?
1.2 Dummy tags: how many tags did you use at each weight? How does the weight compare to the cricket size? How were tags attached to crickets? Rather than having the info about the comparative tags from ATS and lotek (also should include Holohil) in supplemental, put this information in the manuscript. This is important info, especially if someone is planning to use your information to design a tracking study.
1.3 Arena: line 78 ligth should be light. How many arenas did you have?
1.4 Experimental design: first paragraph is confusing. Remember the 1^st^ sentence should be a topic for the entire paragraph. What did the marking mean? Weighing the dummy tag and that superglue was used to attached to the cricket maybe should be included in the dummy tag section? Why did you only record their movement for 10 minutes? Why did you use the same crickets at the same temperature each day? Interesting that you chose to test tags that were heavier than the insects – good! What were you using to measure the impact of tag attachment on behavior? – later text makes it sound like you watched more than one cricket in the arena at a time. That is not clear here. Why did you do that?
1.5 Transforming visual data into numerical data: trajectory refers to a projectile. Haven’t provided enough background information for this section to make sense. What was recorded? What were the positions? What behaviors did crickets perform during their 10 minute observation period? What are the “unique locations”? should include camera information in the experimental design section. I think the second paragraph could be said more briefly and combine it with the information about your team’s software. Why didn’t you use etho vision?
1.6 Statistical analysis: I would include the variables that were measured earlier in the section, not just in the statistical analysis section. Would include a more descriptive definition in table 2 for each property name.

Results

Overall: Instead of trajectory I’d say step or movement length. Trajectory refers to a projectile and makes me think of the overall distance rather than the distance of one step of movement. Give specific examples and descriptions of your results. Mean or range in the measures. Rather than just saying significantly different, include the values. Crickets in the light, medium, and heavy categories moved xx, yy, and zz, cm, respectively. This will show if they are different because the ones in the heavy category didn’t move at all or if they moved, but just less than the other categories. Figures should indicate where statistical significance is present. The text should be able to stand alone without the figures and the figures should be able to stand alone without the text. Then when you have them together the reader should understand the whole picture. This needs to be done for every section of the results. Currently, the results section is very vague.

Discussion

Why didn’t you use etho vision – it shows to work well for studying insect movement with video? Did you have more than one cricket in the arena at one time? Why? Wouldn’t you think that the presence of other crickets could influence their behavior? What about when the crickets were housed over night between their 10 minute observation periods? I would think you would want them to be held in isolation to control their interactions and experiences. What is your justification for your choices? Why is it better to conduct trials with more than one individual at one time?

The first paragraph feels very out of place. I would probably put that information in the introduction?

Video tracking software: this information should be included as justification for the methods you selected not in the discussion

Effects of tagging on the movement of *Gryllus locorojo*:

This should be the start of your discussion. Everything prior to this should find a new home in the intro/methods sections

It feels like a confusing story when temperature is included. Why did you use this as a variable? Is it a biologically relevant question? Why? How would this impact studies in the field?

Discussion needs to be filtered down to tell a clear and concise story. Currently it feels like it is bouncing between ideas and I’m having a hard time trying to identify the point you are trying to make.

Very cool to see even light tag effect distance traveled – I would put more emphasis on what happened because of the tags and not what happened because of temperature. If someone is conducting a field study, the insects will be under field environmental conditions… so I’m missing the point of the temperature variable.

What is the conclusion? Could tags of these weights be used for crickets? Why do you think that?

What would be “negative effects” – can you quantify them so that we can determine when a tag is too heavy for an insect?

The general thoughts section should be integrated into an overall discussion. Right now it is very disjointed.
