# Supplementary material for: Estimating the effect of tracking tag weight on insect movement using video analysis: A case study with a flightless orthopteran
Source: PLoS One. 2021 Jul 22;16(7):e0255117. doi: 10.1371/journal.pone.0255117 (PMC8297838; doi:10.1371/journal.pone.0255117)
Supplement: Attachment — Submitted filename: PONE-D-21-05023_R1_reviewercomments.docx [file pone.0255117.s007.docx]

Abstract: I am still missing the reason why temperature was included in this experiment; it is explained in the introduction, but should also be included in the abstract

Introduction: My concerns were addressed. Could benefit from some tighter language (i.e., make it a bit less wordy – get to the point faster), but no reason to reject the paper.

Line 6: rwith should with with

Line 10: what do you mean by units?

Methods: My concerns were addressed.

Results: Your response is appropriate and the tables with shading address my concerns.

Discussion: My concerns were addressed

Line 347: found should be find
