# Supplementary material for: Estimating the effect of tracking tag weight on insect movement using video analysis: A case study with a flightless orthopteran
Source: PLoS One. 2021 Jul 22;16(7):e0255117. doi: 10.1371/journal.pone.0255117 (PMC8297838; doi:10.1371/journal.pone.0255117)
Supplement: Attachment — Submitted filename: responses.rtf [file pone.0255117.s008.rtf]

Reviewer #1:
You have not dealt with my primary concern about confounding temperature treatments with time in the arena. To be fair, I was not as clear as I could have been about describing the problem that I see. Based on the description of the experiment, each group of test animals is tested in the arena for 3 days in a row, first at the low temperature, then the medium temperature, then at the high temperature. So each group, on day three, has already been in the arena on two previous days. As such, time in the arena is confounded with temperature. I pointed out what I see as a potential solutions to the problem in my original review. You chose to just re-describe the methods. Given the way the paper is currently written I don’t believe you can distinguish the effects you attribute to temperature from effects due to days in the arena.
We acknowledge that we possibly misunderstand the reviewer's primary concern, and thus our previous response may not be clear. However, as we stated in the previous response, we treated temperature as an independent variable, but we should emphasized in the response that each group was recorded in the same temperature for three days in row (e.g., in low temperature - group 1 was recorded three days in row, group 2 was recorded three days in row, …). We tried to describe this approach more clearly in a previous revision of the manuscript in section Experimental design, but it seems that our explanation is still not clear, so we alter the text, mainly editing the last paragraph of the subsection.
If you adjusted the p-values using the Benjamini and Hochberg method using something like the p.adjust R function and type = “BH”, then the citation should be Yekutieli & Benjamini (1999;
https://www.sciencedirect.com/science/article/abs/pii/S0378375899000415
The paper you cite does not adjust p-values, just describes how to adjust the acceptable p-value threshold.
Accepted. We used the “fdr” (“BH”) method in p.adjust function, and changed the reference to Yekutieli & Benjamini (1999) as suggested.


Reviewer #2:
Abstract: I am still missing the reason why temperature was included in this experiment; it is explained in the introduction, but should also be included in the abstract
We agree, thank you for your valuable comment. We have added this information also to the Abstract.
Introduction: My concerns were addressed. Could benefit from some tighter language (i.e., make it a bit less wordy – get to the point faster), but no reason to reject the paper.
The extensive introduction is the result of previous review edits, which heavily improved the readability and flow of this section. Although we reduced the first paragraph, the introduction expanded as we moved the blocks of text from other sections of the manuscript. We tried to be as concise as possible, but at the same time cover the basic information in the individual paragraphs in this order: tracking tag background - types and weights; pitfalls of tag attachments; context-dependent effects and temperature; methods of evaluation of the effects; our research - how differs from previous and what we want to answer. We consider the text in its current form to be balanced. We agree with the opponent that it is a bit wordy, but the topic is quite complex and we want to get the reader into the story in this way. 
Line 6: rwith should with with
fixed
Line 10: what do you mean by units?
replaced with “less than 1 mg”
Methods: My concerns were addressed.
Results: Your response is appropriate and the tables with shading address my concerns.
Discussion: My concerns were addressed
Line 347: found should be find
fixed
