# Supplementary material for: Estimating the effect of tracking tag weight on insect movement using video analysis: A case study with a flightless orthopteran
Source: PLoS One. 2021 Jul 22;16(7):e0255117. doi: 10.1371/journal.pone.0255117 (PMC8297838; doi:10.1371/journal.pone.0255117)
Supplement: Attachment — Submitted filename: responses.rtf [file pone.0255117.s009.rtf]

TIitle: Flightless needs to be added to the Title. Probably the best place is before 'orthopteran', as in 'with a flightless orthopteran'
accepted
Abstract: add 'flightless adult' before 'crickets Gryllus locorojo'

accepted

l. 96 change 'rises' to 'raised'

fixed

l. 206 a permanent repository, such as DRYAD, for your tracking application will need to be designated. Individual websites are not considered permanent.


l. 349 change 'significant affection' to 'significant effects on'

fixed

l. 372 you might also be interested in effects of weighting on dispersal of Anartia fatima doi: 10.3390/insects9030107
You do not mention the effect of changes in position of center of body mass, which is likely to be particularly important for tagged flying insects. 

Thank you for this suggestion. We added this information to the end of the „limitations“ paragpraph, linking it with fact that our study is limited on flightless species, and with flying species there are possible pitfalls of tag attachment.  We thing it fits in this paragraph better and keeps the consistence of Discussion (in proposed paragpraph we discuss only the weight effect in relation to our study).

l. 400 change 'affection of' to 'significant effects on'

accepted

l. 471 change 'brake' to 'break'
fixed
